# Behavioural response of prey to repeated attacks by non-coordinating predators

**DOI:** 10.1038/s41598-025-05946-6

**Published:** 2025-07-02

**Authors:** Siddhant Mohapatra, Pallab Sinha Mahapatra

**Affiliations:** Department of Mechanical Engineering, Indian Institute of Technology Madras, Chennai, Tamil Nadu 600036 India

**Keywords:** Biological physics, Computational biophysics

## Abstract

Collective behaviour is a ubiquitous phenomenon entailing the emergence of fascinating pattern formations in organisms. Reduction of predation risk is presumed to be a major factor contributing towards the evolution of such behaviour. However, the effect of persistent attacks by multiple predators on the behavioural response of the prey remains largely unexplored. The current work aims to address this issue using an agent-based approach employing an underdamped Langevin model. A continuous transition in prey response from a cohesive escape to split-and-escape is discussed with respect to the angular configuration of the predators before the attack. The statistics show that the attack on the nearest prey is the most successful pursuit strategy, while alternative strategies, such as attacking the centre of the group, have conspicuous ancillary effects, such as group splitting. A long-term temporal study of the system indicates a counter-intuitive faster decay of prey numbers at higher intensity of prey coordination, hinting at possible excess alignment and its detrimental effects in the case of successive predator attacks. The effect of predation is found to be non-additive even if non-coordinating predators are considered, highlighting the non-scalability of predator-prey systems and urging further scrutiny of the dynamics of group hunting in such systems.

## Introduction

Collective behaviour is a ubiquitous and fascinating natural phenomenon observed in a wide range of organisms across size scales. From an evolutionary standpoint, the dilution of predation risk remains a plausible explanation for the origin and development of such phenomena. Works by Ioannou et al.^1^ showcasing natural predators preferentially attacking isolated over aggregated virtual prey, and Olson et al.^2^ propositioning that predator confusion alone can warrant swarming behaviour, hint at the link between the predation risk and the evolution of collective motion. Apart from promoting predator confusion, collective behaviour can also reduce the individual burden of vigilance against predators^3–5^. The early detection of predators’ approach and fast and optimised information transfer due to flocking allow the prey to perform well-coordinated movements, which have been a focal point of research over the past decades. Magurran and Pitcher^6^ reported a wide array of collective evasive behaviour (most commonly compact, inspect and avoid) undertaken by minnows (*Phoxinus phoxinus*) and the frequency of their occurrence in response to pike (*Esox lucius*) attacks. Similarly, Storms et al.^7^ documented the collective escape patterns, such as blackening, wave event, split, and merge, to name a few, in a starling (*Sturnus vulgaris*) flock under the threat of predation from a falcon. They examined the circumstances surrounding the incidence of each evasive measure and connected them to the threat intensity of the falcon attack (including the speed of the attack) and the state of the flock before the attack. Papadopoulou et al.^8^ analysed trajectories of pigeon flocks chased by a robotic falcon and developed a model to demonstrate the relationship between the propensity for turning and distance from the predator, leading to an effective collective turn negotiation. Domenici and Batty^9^ discussed how schooling assists herring (*Clupea harengus*)  in rectifying their trajectories while turning in response to an acoustic disturbance. Palmer and Packer^10^ investigated the response of wild impala, zebras and wildebeest to high-resolution life-sized images of common African predators. They reported that the nature and intensity of collective escape behaviour is species-dependent and is a function of the predator’s hunting style and the prey’s risk perception.

Taking a broad overview of animal behaviour-based studies, a fair amount of literature involves observations in the wild^10–13^ as well as experiments in controlled environments^14–17^. Such hands-on approaches help establish empirical relations pertaining to the social behaviour of organisms. Computational models can then be employed to identify the underlying mechanisms of such collective behaviour. Agent-based modelling has been a prevalent technique for simulating collective behaviour in organisms, as they allow researchers to not only analyse individual-level details but also tweak and test possible interaction rules. Over the past few decades, researchers have developed numerous mathematical models^18–21^ to explain how living and artificial active matter self-organise and dynamically maintain the structural integrity of the collective. Agent-based models have been particularly useful in faithfully recreating and analysing collective patterns^22,23^ and motion features observed in natural conditions^8,24–26^.

Interactions between adversarial species, aptly named predator-prey interactions, are a major research focus in the biophysics community due to the degree of complexity involved. A significant section of the existing studies have focused on the transmission and the role of visual, olfactory and auditory cues when predators and prey are in proximity^27–30^. Barring a handful of studies^31–36^, the majority of the extant literature (especially computational) is concerned with a single predator interacting with a prey group rather than successive or concerted predator attacks. However, experimental observations have highlighted the ubiquity of group hunting in numerous ecosystems^37–40^. The reviews by Sih et al.^41^, Bailey et al.^42^, and more recently, Hansen et al.^43^ accentuate the benefits of group hunting while emphasising the lack of comprehension of the underlying physics involved in such scenarios. The attack patterns of the predators have also been explored to a limited extent in literature^10,44–47^; however, there is a scarcity of studies highlighting the effect of predator attacks using a combination of different strategies.

The current work focuses on explaining the effect of repeated predator attacks on a prey group using an agent-based model. Emphasis has been placed on analysing the prey response to different strategies and positional configurations of the predators during the attack, as well as the intensity of prey coordination. The detrimental consequences of excessive alignment among the prey and its relevance in the case of repeated predator attacks have been deliberated upon. The impact of two independently hunting predators has been discussed and contrasted with a single predator system, highlighting the non-additive nature of predation. The next section describes the behavioural response of the prey to the predator attacks, followed by a quantitative take on the long-term and the transitory system behaviour and a comparison of the hunting performance of a single predator and two non-cooperative predators on the same prey group. The subsequent section presents the details of the numerical model along with a description of the systemic parameters.

## Results

The current work employs agent-based simulations to study the behavioural response of a group of 506 prey to two predators in a periodic domain. The focus of the study is on macroscale animals, which display collective behaviour in the inertial regime. Therefore, under-damped dynamics have been used to describe the motion of the agents. An analysis has been carried out to determine the precursors affecting the nature of the response. This is followed by a quantitative take on the long-term and transient behaviour of the predator-prey system, taking into account the choice of targets of the predators and the impact of persistence and asynchrony of the predator attacks. The last subsection contrasts the performance on a per-head basis of a lone predator against that of two asynchronously attacking predators under similar conditions.

**Fig. 1:**
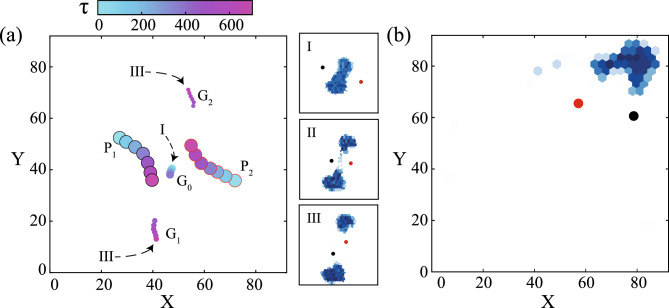
The prey often respond to an attack by predators ($P_1$ and $P_2$) by two mechanisms; the first is shown in panel (**a**), where the prey group ($G_0$) splits into smaller groups, $G_{1}$ and $G_{2}$ (the groups are represented by their centre of mass, and the colour bar shows the time progression of the event, where $\tau =\frac{t}{\sqrt{d_{pr}/\beta _{pr,e}}}$ is the non-dimensional time), and the second is shown in panel (**b**), where the prey group stays cohesive and collectively moves away from the approaching predators. In panel (**a**), the predator positions are tracked alongside the centroids of the groups before and after splitting. Panels (a-I) through (a-III) depict the group-splitting phenomenon through time-lapse prey density plots.

### Collective choice: stay cohesive or split

In a simulated attack by the predators, the prey exhibit a collective response mostly by one of two mechanisms: the first involves moving away from the attacking predators while maintaining group cohesion, while the other involves splitting of the prey group into smaller divisions and escaping. Evidence of such behaviour in nature has been documented a priori in minnows^6^, teleosts^48^, herring^49^ and more. The two types of prey response are elucidated in Fig. 1, the split response in Fig. 1(a), and the group escape while staying cohesive in Fig. 1(b). In Fig. 1(a), the predators are denoted by $P_1$ and $P_2$, while the prey groups are denoted by their centre of mass $G_{i}$ (where *i* is the group designation). The time progression ($\tau =t/\sqrt{d_{pr}/\beta _{pr,e}}$) of the response is shown through the attached colourbar and has been non-dimensionalised with the time a prey agent takes to traverse its own diameter at escape acceleration. The path traced by the two predators, $P_1$ and $P_2$, shows the approach from opposite directions towards the undivided group $G_0$ (also see Fig. 1(a-I)). As the predators move closer to group $G_0$, the prey attempt to move in a transverse direction to the predators’ line of approach. Based on the proximity to the respective predators, the group’s attempt to escape causes a constriction at the location nearest to both the predators (hourglass shape; see Fig. 1(a-II)). The constriction continues narrowing, and the initial group ($G_0$) eventually splits into smaller groups ($G_1$ and $G_2$), which start moving in opposite directions (as depicted from the colour profile), and the predators follow the group in their immediate neighbourhood (see Fig. 1(a-III)). The grouping of prey has been carried out using the DBSCAN algorithm, the details of which are mentioned in the subsequent paragraph. Alternatively, the prey group remains cohesive and performs a directed motion away from the predators’ approach (see Fig. 1(b)). The uni-directional movement of the prey is a consequence of the lower angular difference in the predators’ approach towards the prey group. For more details, please refer to the Supplementary Movie SV1.

**Fig. 2:**
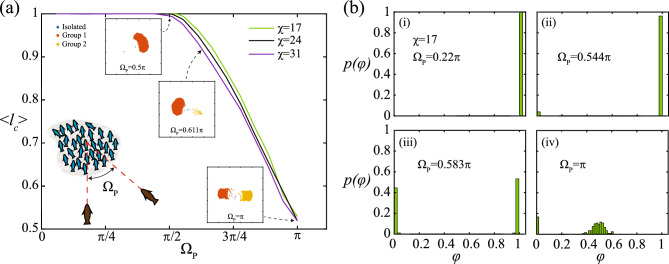
The figure showcases the dependence of the prey response (to stay cohesive or to split) to the relative angle of predator attacks $\Omega _P$ (see inset schematic for $\Omega _P$ in panel (**a**)). Panel (**a**) delineates the effect of $\Omega _P$ on the average fraction of agents constituting the largest prey group after the response (hereby, referred to as group size) denoted by $l_c$. Irrespective of prey coordination strength $\chi =\frac{C_v d_{pr}}{m_{pr}} \sqrt{\frac{d_{pr}}{\beta _{pr,e}}}$, $\langle l_c \rangle$ decays monotonically with $\Omega _P$, denoting a transition from a cohesive escape response to a split-and-escape response. The insets in panel (**a**) are representative snapshots of the system post-response to illustrate the transition process. Panel (**b**) charts the probability of finding a group of size $\varphi$ at different $\Omega _P$ for $\chi =17$. (The group size has been normalised against the initial number of prey agents in the simulation. Fifty realisations have been carried out for each value of $\Omega _P$ and the data used for calculating $\langle l_c \rangle$ and plotting the probability distributions).

Whether the prey perform a splitting response or escape cohesively is closely related to the group’s heading and the predators’ relative approach. To examine the dependence of the group response on the latter, simulations involving a single attack from both predators moving towards a prey aggregation (of $N=506$ agents) at different relative attack angles $\Omega _P$ (see schematic of Fig. 2(a)) has been carried out, and the ensuing prey response recorded. The fraction of agents constituting the largest prey group in the domain following the predators’ attack has been computed as $l_{c}$ (normalised against the initial prey aggregate numbers, i.e., $N=506$) using a DBSCAN (Density-Based Spatial Clustering of Applications with Noise) algorithm in MATLAB by considering the neighbourhood search radius as $r_{alg}$ and the minimum number of agents within a neighbourhood to be identified as a cluster set at $1\%$ of initial prey numbers *N*. With an increase in $\Omega _P$, the prey group exhibits a transition from a cohesive escape response to a split-and-escape response. The largest group size $l_c$ has a monotonically decreasing relation with $\Omega _P$ (see Fig. 2(a)). The insets of Fig. 2(a) illustrate representative snapshots of the system after the predators’ attack for different $\Omega _P$. In all three images, the prey group coloured red (designated as Group 1) is the largest prey group in the system. Predators attacking at higher $\Omega _P$ tend to not only lead to group splitting but also influence the distribution of the agents into the resulting groups of the split. At $\Omega _P=\pi$, the distribution of the prey agents into the two resultant groups stands almost equal $\left( \langle l_c \rangle \approx 0.52\right)$. Such behavioural transition from cohesive to splitting response is true irrespective of the prey coordination strength $\chi$ (which is the relative strength of the alignment force to the self-propulsion force in the escape state; $\chi = \frac{C_v d_{pr}}{m_{pr}}\sqrt{\frac{d_{pr}}{\beta _{pr,e}}}$; more details in the Methods section). However, at higher coordination strength ($\chi =31$), the transition to splitting response occurs at a marginally lower $\Omega _P$ compared to that of lower prey coordination strength, as can be seen in Fig. 2(a) ($\langle l_c \rangle$ starts decreasing below 1 earlier for $\chi =31$ as $\Omega _P$ increases). This denotes that higher coordination among prey serve as a weak promoter of the splitting response to predator attacks.

Figure 2(b) provides the statistics of the post-response group sizes $\varphi$ (normalised against the initial number of prey agents in the system, i.e., $N=506$) at different $\Omega _P$ for $\chi =17$. The probability of finding a group of size $\varphi \approx 1$ is 1 in Fig. 2(b-i) ($\Omega _P=0.22\pi$), indicating the prevalence of only the cohesive escape response. As the relative angle of predators’ attack increases, a small fraction of prey are found to split (sometimes) from the initial aggregate (see Fig. 2(b-ii); $\Omega =0.544\pi$), marking a transition to a split response (albeit, hardly perceptible). With further increase in $\Omega _P$, however, the dynamics start changing drastically in favour of the split response; at $\Omega _P=0.583\pi$, small groups of prey tend to split away from the larger group more often in the case of predator attacks. At very high $\Omega _P$ ($\Omega _P=\pi$), the distribution of the prey groups becomes concentrated between $\varphi =(0.4,0.6)$, indicating comparable group sizes post-split (as seen in the inset of Fig. 2(a); $\Omega _P=\pi$). It is to be noted that isolated agents (agents with neighbours numbering less than $1\%$ of *N* within the neighbourhood of $r_{alg}$; coloured blue in the representative insets of Fig. 2(a)) are not considered while plotting the probability distributions in Fig. 2(b), therefore, the values of $p(\varphi )$ on the lower end of the $\varphi$ scale are due to the occasional formation of small prey groups during the splitting process.

**Fig. 3:**
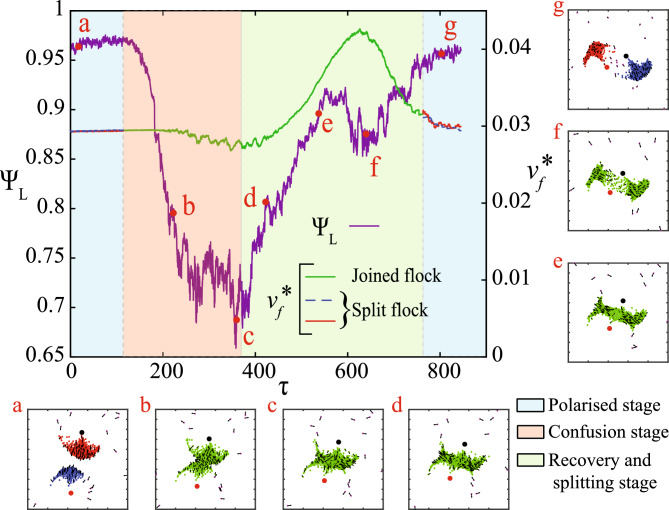
The local order parameter $\Psi _L$ (violet solid line) and the non-dimensional average speed of the group(s) $v_f^*$ are plotted against time $\tau$ during a merge and subsequent split phenomenon, with snapshots of the system illustrating the headings of the agents at different times. In the first stage (region shaded light cyan; the polarised stage), there are two distinct polarised groups (inset (a), agents coloured red and blue, respectively) moving in a path for imminent collision. After the collision, a united group is formed (inset (b), agents coloured green), and there is a temporary reduction in the local order (region shaded pale red; the confusion stage). The insets (b) and (c) show the progressively dissimilar orientations of the colliding groups during the confusion stage. The united group eventually recovers from the confusion (indicated by recovering $\Psi _L$; region shaded pale green) due to alignment interactions (inset (d)) until opposing predation pressures cause stretching of the group (marked by an increase in $v_f^*$ from typical levels) in a direction transverse to the line of the attack (insets (e) and (f)). The narrowing of the constriction eventually leads to the splitting of the group into two, each of which now exhibits polarised motion, thereby completing the cycle (inset (g)). (The red solid line and the blue dashed line in $v_f^*$ correspond to the two polarised groups, while the green solid line corresponds to the united flock shown in the insets; $\chi =17$).

### Merge and subsequent split: an effect of repeated predator attacks

The previous subsection focuses on the more frequently observed responses of a prey group to a simulated attack by a predator duo. A somewhat rarer feature that is also observed is the merging of two or more prey groups due to the attraction force among the prey (details in the Methods section). The merge phenomenon is biologically significant as it promotes the formation of larger groups, thereby diluting the risk of predation further as hypothesised by the many-eyes theory (which states that the larger the group, the lesser the individual burden of vigilance against predators, and the faster the response to perturbations). However, due to the presence of multiple independent predators, such phenomenon is often followed by a splitting response, which highlights the dynamic nature of the prey response. The merge and split prey response (as the overall process is hereby referred to) is characterised by employing the local order in the prey group $\Psi _L$ and the average speed of the prey group(s) $v_f^* = \frac{1}{v_0}\left\| \frac{\sum _{pr \in G} \mathbf {v_{pr}}}{N_{G,pr}}\right\|$ (*G* being the prey group and $N_{G,pr}$ being the number of agents in the group). $v_0=\sqrt{d_{pr}\beta _{pr,e}}$ is the normalisation factor for the speed and is calculated as the speed attained by a non-interacting agent having traversed across its own length (diameter) with escape acceleration $\beta _{pr,e}$. $\Psi _L$ is calculated from the degree of agreement of the orientations of all unique neighbouring live prey pairs in the domain (see Eq. 1).

1 $$\begin{aligned} \Psi _L = \frac{1}{\mathbb {N}} \sum _{i} \sum _{j > i} \frac{\mathbf {v_i} \cdot \mathbf {v_j}}{\Vert \mathbf {v_i} \Vert \Vert \mathbf {v_j} \Vert } \mathcal {H} \left( r_{alg} - \Vert \mathbf {r_{ij}} \Vert \right) \end{aligned}$$

where, $\mathbf {v_{pr}}$ is the velocity of prey agent *pr*, $\mathbb {N}$ is the number of unique neighbour pairs among the live agents, and $\mathcal {H}$ is the Heaviside function. A highly polarised group would consist of agents moving in almost the same direction, therefore, $\Psi _L \approx 1$. On the other hand, the agents in a completely disorganised group would have their headings distributed uniformly across $[0, 2\pi )$, resulting in local order $\Psi _L \approx 0$.

Figure 3 demonstrates the variation of the local order parameter $\Psi _L$ and the speed of the groups $v_f^*$ (computed as the average of the non-dimensional speed of the live prey agents in a group) across the different stages of the merge and split phenomenon at lower prey coordination ($\chi =17$) values: a polarised stage (shaded light cyan), a confusion stage (shaded pale red) and a recovery and splitting stage (shaded pale green). The first stage involves two highly polarised groups ($\Psi _L \approx 0.96$; each group carrying out cohesive escape response) moving with an average speed typical of $\chi =17$ on an imminent collision course due to persistent pursuit by their respective predators (refer to Fig. 3 inset (a)). As the two groups collide, it leads to a temporary disruption of order (confusion), indicated by a sharp reduction in the local order parameter $\Psi _L$ (refer to the agent orientations in Fig. 3 inset (b)). The average speed of the united group $v_f^*$ (green solid line) is also negatively affected due to the collision. Eventually, due to the alignment interactions among the prey agents, the local order in the system is recovered (with an increasing trend of $\Psi _L$; refer to the order in the agent orientations from inset (c) to inset (d)). Meanwhile, the two predators approach the united group from opposite directions, compelling the agents to move in a direction transverse to the line of the predators’ attack (see Fig. 3 inset (e)) and leading to a splitting response (note the constriction as seen in Fig. 1(a)). The approach of the predators causes an increase in the average prey speed in the transverse direction as the constriction becomes sparser (see inset (f)), culminating in a complete split of the united group into two highly polarised groups (group speed represented by solid red and dashed blue lines; also see inset (g)). The recovery of the local order $\Psi _L$ does not follow a monotonic trend, with a slight dip around point *f* in Fig. 3. This is a consequence of the prey agents farthest away from the corresponding predators being unable to detect the predator’s presence and, therefore, entering a cruising state, which reduces the agreement of their headings to the agents still in the escape state (inset (f)). The merge and split phenomenon, observed in Fig. 3 , has a unique signature of a transitory reduction in local order, accompanied by an increase in group speed during the recovery stage. The absence of any of the above features indicates the absence of a merge and split response. Merging and splitting, as well as the cohesive escape/split-and-escape, can be observed in systems with a single predator as well^6^; however, in this particular variant, due to the multiple predators, the time associated with the confusion stage during a merge-and-split is a substantial disadvantage for the prey (confusion translates to slower response time and reduced coordinated evasion capabilities). Moreover, the persistent predator attacks make it challenging for large groups to subsist in the domain.

### Quantitative analysis of prey response

The previous subsections give a qualitative overview of the prey response to the predators’ attacks. However, there is also a need to understand the changes in the prey response with respect to the systemic conditions and to assess the efficiency of the predators in capturing the prey under such circumstances.

**Fig. 4:**
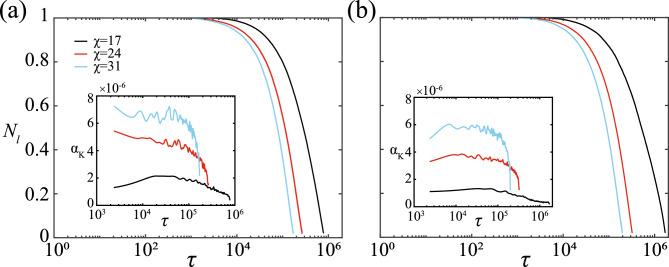
The figure highlights the temporal variation of the fraction of live prey $N_l$ for (**a**) a purely NP strategy and (**b**) a probabilistic NP-MCP combination strategy. The insets illustrate the change in the prey capture rate $\alpha _K$ with non-dimensional simulation time $\tau$. (Note: $N_l$ is calculated as the number of live prey normalised by the initial number of prey in the system *N*, here, 506. Prey capture rate $\alpha _K$ is calculated by computing the slope of $N_l$ considering every pair of consecutive points and then averaging the values over a small moving window.)

**Fig. 5:**
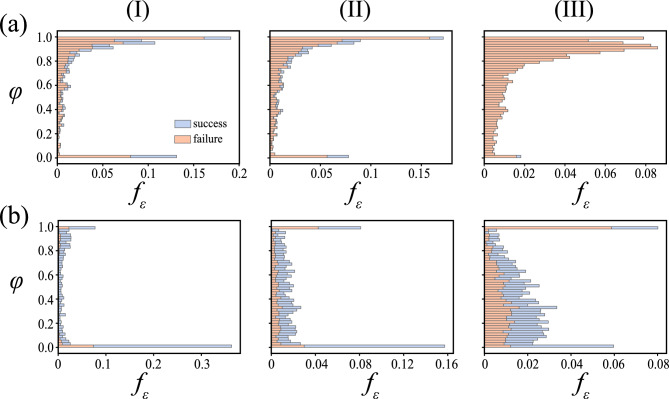
The figure elucidates the fraction of pursuits on prey group of size $\varphi$ (normalised against the total number of prey agents in the system initially, *N*) that end up in success (blue) or failure (orange) $f_\epsilon$, at prey coordination strength (**a**) $\chi =17$, and (**b**) $\chi =31$. Each of the columns represents a different set of attack strategies: (I) a purely NP strategy, (II) a probabilistic NP-MCP combination strategy, and (III) a purely MCP strategy, respectively. ($f_\epsilon$ is computed with respect to the total number of pursuits across all the realisations for a particular attack strategy.)

#### Long-term system behaviour

A predator’s hunting prowess can be estimated by examining its rate of prey capture. Figure 4 depicts the decay in the fraction of live prey in the system $N_l$ (i.e., the ratio of the number of live prey to the initial number of prey *N*) with time due to capture by the predators, while the insets delineate the rate of capture $\alpha _{K}$ (calculated as the slope of $N_l$ and then averaged over a small moving window) of live prey with time at different coordination strengths $\chi$. To note, there is a limited number of prey in the system, and captured prey are not replaced with new live prey. The predators choosing to attack their respective nearest prey (purely NP strategy) leads to a faster rate of prey capture compared to an NP-MCP combination attack (the rate is roughly $20\%$ higher for the purely NP attack). The adverse effect of superfluous coordination can be observed by comparing the $N_l$ curves for $\chi =17$, $\chi =24$ and $\chi =31$. The rate of capture is lower in the case of lower coordination, owing to higher speeds and greater freedom of individual movement (see Fig. S1 of Supplementary Information). Regardless of the individual attack strategies of the predators, the capture rate seems to follow a decreasing trend with time for any prey coordination strength except $\chi =17$ for the majority of the simulation time. The gradual decline in $\alpha _K$ can be explained by a gradual decrease in available live prey numbers. It might also be an artefact of the predation model, which considers a limited hunting time, a refocus time and a satisfaction time (details in the Methods section). A statistical analysis of the temporal dependence of the fraction of live prey $N_l$ is detailed in Sec. SI-1 (Fig. S2 and Tables S1 & S2) of Supplementary Information. When there is an abundance of target prey options for the predators, the predators are successful in capturing the target within the stipulated hunting time, which is evidenced by an ever so slightly decreasing $\alpha _K$ (see Fig. 4 insets) across the majority of $\tau$. However, in the later stages of the simulation (high $\tau$), a rapid decrease in the capture rate is observed owing to the sparsity of live prey in the domain, forcing the predators to conduct a protracted search before pursuit and capture, thereby marking a departure from linearity.

#### Transient system behaviour

The decay in the fraction of live prey in the domain provides a broad idea of the predators’ pursuit capabilities. However, details of the transient effects of parameters, such as prey coordination and predators’ attack strategy, are required for a holistic understanding of the system. This is achieved by using the results of the pursuits as the metric. The general notion in predator-prey systems is that the smaller the group of prey, the easier the hunt^16^. Therefore, it is vital to examine the effect of prey group size on pursuit success. Figure 5 portrays the distribution of successes and failures of attacks on different prey group sizes $\varphi$ (normalised against the initial number of prey in the system *N*) for different predator attack strategies and prey coordination strength.

At low prey coordination strength ($\chi =17$), the majority of the attacks are focused on larger group sizes, although a significant portion of them end in failure. The predators adopting a purely NP attack strategy have the highest probability of pursuit success, with $\approx 28\%$ of all such encounters resulting in successful prey capture, followed by the NP-MCP combination attack strategy at $\approx 17\%$ and the purely MCP attack strategy at $\approx 0.26\%$. The statistics imply that the purely MCP attack strategy is clearly unfavourable for successful prey capture and is rather geared towards reducing the group sizes through splitting, as indicated by the relatively higher number of encounters at mid-to-low group sizes in Fig. 5(a-II) and Fig. 5(a-III) (the NP-MCP combination and the purely MCP strategies, respectively), in comparison to the purely NP strategy (Fig. 5(a-I)). Extant literature highlights predatory inclination towards pursuing isolated prey and prey in small groups^6,16,45^, and such behaviour is also observed in the current work, accounting for $\approx 18\%$ of captures in the case of the predators following purely NP strategy, $\approx 12\%$ in the case of the NP-MCP combination strategy and $\approx 84\%$ in case of the purely MCP attacks at $\chi =17$.

Even when the prey tend to coordinate more strongly (i.e., higher coordination strength $\chi =31$), the purely NP attack strategy still leads to the highest pursuit success rate ($\approx 81\%$) compared to the NP-MCP combination strategy ($\approx 68\%$) and the purely MCP strategy ($\approx 57\%$). The success rate of all three attack strategies increases by $\approx 50\%$ with the increase in $\chi$ (from 17 to 31). It can also be noted that a significant number of attacks take place at very low cluster sizes, i.e., there is a clear predatory bias towards small prey groups and isolated prey, accounting for $\approx 36\%$, $\approx 19\%$, and $\approx 8\%$ of the prey captures in case of the purely NP, the NP-MCP combination and the purely MCP strategies, respectively. The pursuit data at different levels of prey coordination propensity clearly shows that the purely NP strategy is the most effective for non-coordinating or competing predators. However, when the prey coordinate to a greater extent, the rate of success of attacks on smaller prey groups far exceeds that on the larger groups, which highlights the benefits (to the predators) of using a purely MCP strategy intermittently to cause splitting of the large prey groups.

**Fig. 6:**
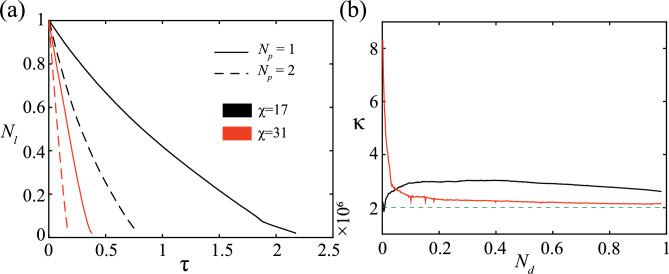
Panel (**a**) portrays the decay of the live prey fraction $N_{l}$ in the case of a single predator following a NP strategy (solid line) and two predators following a purely NP strategy (dashed line) for prey of different coordination strengths $\chi =17$ (coloured black), and $\chi =31$ (coloured red). Panel (**b**) elucidates the variation of $\kappa$, the ratio of the time taken by a lone predator to capture a certain fraction of prey agents $N_d$ to the time taken by two non-coordinating predators for the same fraction. A solitary predator takes much longer to capture prey compared to a predator duo when there is an abundance of live prey in the system (low $N_d$). The disparity reduces as the availability of live prey starts diminishing. Nevertheless, the time taken by a single predator to capture any fraction of prey is always found to be more than twice that required by two predators (i.e., the effect of predation is non-additive). (Note: The fractions $N_l$ and $N_d$ are calculated as the number of live prey and number of captured prey in the domain, normalised against the initial number of prey *N*).

### On the non-additive effect of predation

The results so far have dealt with understanding the prey group response when attacked by two independently moving predators. Prey behaviour such as cohesive escape or splitting can be realised in the presence of a single predator as well and has been extensively studied in extant literature^7,8,45,50^. Therefore, it is crucial to discern the differences in prey response in the presence of two predators compared to that of a solitary predator. Figure 6(a) illustrates the temporal variation of the fraction of prey alive $N_{l}$ when attacked by a single predator following the NP hunt strategy and compares it to that of two predators following a purely NP strategy under the same systemic parameters (such as $\chi$). The solid line corresponds to the case of a lone predator, while the dashed line corresponds to the case of two asynchronous predators. The decay of $N_l$ is slower at $\chi =17$ (coloured black) compared to $\chi =31$ (coloured red), in line with Fig. 4. Quite obviously, the single predator requires more time than that required by two predators to capture the same fraction of prey. However, what’s not so intuitive is that the single predator takes more than twice the time required by the non-coordinating predator pair. A metric $\kappa = \frac{\tau \left( N_d; N_p=1\right) }{\tau \left( N_d; N_p=2\right) }$ has been defined to account for this time disparity and has been delineated in Fig. 6(b), $N_p$ referring to the number of predators. $\kappa$ can be described as the ratio of the average time taken by a single predator to that required by a non-coordinating predator duo to capture a certain fraction of prey agents $N_d$. For any fraction of captured prey agents $N_d$, $\kappa$ has a value greater than 2, highlighting the non-additive effect of predation. At high prey coordination strength ($\chi =31$), during the initial stages of the simulation (low $N_d$), $\kappa>> 2$, connoting a highly non-additive nature of predation. With more prey ending up captured (as $N_d$ increases), $\kappa$ declines and settles at a value slightly greater than 2. A similar behaviour is observed at low prey coordination ($\chi =17$), with the exception of the high extent of non-additive nature at low $N_d$. Nevertheless, the low extent of coordination permits the prey to move at higher speeds (see Fig. S1 of Supplementary Information) and evade a single predator with relative ease, which is evidenced by $\kappa$ being close to 3 for a significant set of values of $N_d$.

## Discussion

The current work conducts a systematic evaluation of the collective prey response to repeated attacks by two non-coordinating predators. The organisms (the prey and the predators) are modelled as agents, experiencing certain drives (expressed in terms of forces). The prey respond to the attacks either by escaping while maintaining cohesion or by splitting and escaping from the pursuing predators. The predators follow the “pure pursuit” approach, where the agent dynamically chases the moving prey by accelerating towards its last known location. Alternatively, more complex schemes of pursuit, such as deviated pursuit and parallel navigation^46^ can also be used. Another point to note is the proclivity of the predators to pursue the prey persistently, also known as coursing, contrary to an ambush attack^10^. The current work also assumes that the predators pursue the prey by sight rather than relying on an ambush tactic. Additionally, the predators are also assumed to be non-consuming (i.e., when the prey are captured, they still exist in the system as dead agents), although it is observed that consuming predators lead to almost identical long-term behaviour as non-consuming predators (see Fig. S3 of Supplementary Information). The prey evasion, as well, occurs using an avoidance rule: accelerating away from the predator’s last known position. In some organisms, the prey might take an alternate protean approach to evasion (i.e., a stochastic direction of evasion)^51^. However, a recent study by Szopa-Comley and Ioannou^52^, involving robotic prey and natural predators, has challenged the efficacy of the protean approach in the long-term dynamics of such systems.

Visual cues have been reported to form a major basis for risk perception in certain predator-prey pairs^29^. Therefore, our model considers the interactions among the agents based purely on visual cues. Visual occlusion (due to other agents obstructing the line of sight) has not been incorporated into our model for the sake of simplicity. The debate on the practicality of aposematism (as a purported defence mechanism) on predation viability advocates the importance of visual cues in prey selection^53,54^. In the purview of such significant promoters of visual cues, other sensory inputs, such as auditory and haptic, have been neglected in the current work. Nevertheless, in certain predator-prey pairs, auditory inputs have been found to be the major source of predation risk^55,56^. Additionally, the model does not account for environmental factors such as the heterogeneity in the environment (including the presence of obstacles and turbidity in the medium), which can have an effect on the collective dynamics of the prey^57–59^. To account for such complexities, one can modify the current model to include visual occlusion explicitly, similar to^60,61^.

Every agent in the current model is subjected to a self-propulsion force (evasion drive for the prey and pursuit drive for the predator), an attraction force, a friction drag, a short-range separation force and an alignment force. The alignment force is the most vital among these forces, as it promotes schooling in prey rather than swarming. However, at high coordination among the prey, the speed of the prey diminishes, resulting in sluggish motion. A possible cause for this slowdown could be the nature of the alignment force used in the model, which aligns both the orientation and the magnitude of the velocity, as opposed to the Vicsek model^20^ (which aligns only the orientation). This underdamped approach to implementing alignment can cause retardation to any sudden change in agent velocity in a direction dissimilar to the velocity of the neighbouring agents. In other words, the retardation can be interpreted as a consequence of alignment for the prey and discourage individualistic behaviour such as flash expansion. In order to simulate flash expansion in prey groups with the current model, the prey coordination has to be reduced to minuscule values.

The size of the initial prey group can also affect the efficacy of the collective escape behaviour of the prey in response to predation. Larger groups are usually deemed safer owing to the dilution of predation probabilities and better detection abilities (higher number of vigilant entities). Figure S4 of Supplementary Information sheds light on the variation in prey capture rates considering different initial group sizes and shows good agreement with the above-mentioned hypothesis.

Although the predator-prey dynamics involving a single predator and one or more prey are well-documented in the extant literature, the complexity of the dynamics scales precipitously even with an additional predator. The effect of multiple predators is clearly non-additive. Simulations with more than two non-interacting (except volume exclusion) predators also yield similar qualitative results as reported in the case of two predators (see Fig. S5 of Supplementary Information). Even discounting the combinatorics of the predators’ target selection strategies, parameters such as the relative angle among the predators during the attack and the synchronisation (phase) dynamics of the predators’ attacks play a major role in the success of prey capture. Depending on the relative angle of the predators’ attack, the prey exhibit a continuous transition from a cohesive escape response to a splitting response. Coordination among prey is reported to help improve the odds of prey survival in the presence of a solitary predator^50^. However, the coordination benefits are not as evident when multiple predators are involved. A slight phase difference (i.e., mismatch) in the predators’ attack timings can be detrimental for the prey, possibly even to the extent that the group escape response to the first attack ends up benefiting the predator attacking second. In phenomena such as merge and split, the merging of the multiple prey groups causes a period of confusion for the prey regarding the direction of movement of the united group. A well-timed attack by a predator can take advantage of such a scenario, leading to higher prey capture. The role of asynchrony is crucial, as well, in determining optimal attack patterns in systems with multiple predators, as has been carried out for a single predator in existing literature^35,45^. As the disturbance in the local order of the prey group caused by every predator attack outlives the attack duration itself, the frequency of attacks also has far-reaching effects on the efficacy of prey evasion behaviour. Theibault et al.^36^ reported the improvement in hunting success per predator with successive attacks on a prey aggregate from experimental observations, while Lett et al.^35^ detailed the detrimental effects of an increasing frequency of predator attacks on the flash expansion characteristics of the prey group using an attraction-alignment-repulsion model.

Group hunting tactics are another way for predators to increase their hunting success, as has been reported in African lions^37,38^, African wild dogs^39^, and killer whales^40^, among others. The current work examines the efficacy of different predators’ attack strategies, highlighting the need of attacking the most central prey intermittently to divide the group and attacking the nearest prey otherwise to maximise chances of prey capture. When used in tandem in a synchronised fashion, a combination of these attacks can nudge the prey capture success rates even higher (as is observed in the works on group hunting).

The model explained in the current work provides us with a framework to simulate and comprehend the transitory and the long-term behavioural dynamics of a prey group when attacked by multiple non-coordinating predators. A subsequent course of action would be to understand the mechanism of group hunting, which remains rather ambiguous despite the numerous observations in the wild. A major advantage of using an agent-based model is the ease of fitting the model to different predator-prey pairs based on their physical and social attributes and available experimental data. However, as mentioned earlier, the model requires certain underlying assumptions in its formulation, which leaves room for improvement.

## Numerical methodology

The current work employs an agent-based modelling approach, wherein all the entities are assumed to be agents placed in a periodic domain. The motion of the agents is governed by underdamped Langevin dynamics with non-negligible agent inertia. In this work, the agents can be classified into two categories: a prey agent and a predator agent, with their specific characteristics discussed below.

### Prey agent

The governing equation for a prey agent *pr* consists of a self-propulsion term, a short-range pairwise separation term, an alignment term, a friction drag, and a long-range attraction term (see Eq. 2).

2 $$\begin{aligned} m_{pr}\mathbf {\ddot{x}_{pr}} = \mathbf {F_{s,pr}} + \mathbf {F_{f,pr}} + \mu \left( \mathbf {F_{p,pr}}+\mathbf {F_{c,pr}}+\mathbf {F_{a,pr}}\right) , \end{aligned}$$

where, $m_{pr}$ and $\mathbf {x_{pr}}$ are the mass and the position of the prey agent, respectively, $\mathbf {F_{s,pr}}$ is the pairwise separation force, $\mathbf {F_{p,pr}}$ is the self-propulsion force, $\mathbf {F_{c,pr}}$ is the alignment force, $\mathbf {F_{a,pr}}$ is the attraction force, $\mathbf {F_{f,pr}}$ is the frictional force and $\mu$ determines whether the prey is alive ($\mu =1$) or captured ($\mu =0$).

**Fig. 7:**
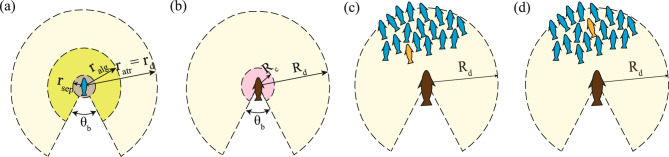
The zone-based classification of forces for (**a**) the prey and (**b**) the predator agents are elucidated. Notations for the predator are c: capture, d: detection; while for the prey, sep: separation, alg: alignment, atr: attraction, and d: detection zones respectively. $\theta _b$ is the blind spot in the vision of the agent. The different strategies that the predator can adopt while attacking prey groups are showcased here: (**c**) nearest prey attack and (**d**) most central prey attack. (The blue prey are possible targets, the orange prey is the selected target prey, and the predator is coloured brown.)

The model considers a zone-based classification around the prey agent for the action of the forces. Every prey agent has a separation zone, an alignment zone, and an attraction zone, based on which the forces act on the prey agent (see Fig. 7(a)). The separation force acts only within the limits of the separation zone ($0 < r \le r_{sep}$), and the alignment force acts in the alignment zone sans the separation zone ($r_{sep} < r \le r_{alg}$), while the attraction force acts in the area between the alignment zone limits and the attraction zone limits ($r_{alg} < r \le r_{atr}$). Apart from these zones, the prey agent has a detection zone encompassing the entire zonal area ($0 < r \le (r_{atr}=r_d)$), where the agent can detect the presence of the predator. Assuming the sensory input to be predominantly visual^29^, a blind angle $\theta _{b}$ is considered symmetrically about the direction opposite to the agent’s heading (refer to Fig. 7(a)).

The pairwise separation force is a short-range repulsive force that prevents the overlap of agents^50^ and is modelled as a Hertzian contact force per unit length between two cylinders overlapping along their length dimension (as the agents are modelled as soft disks). The force acting to separate an agent *i* from any agent *j* in its separation zone is presented in Eq. 3.

3 $$\begin{aligned} \mathbf {F_{s,i}}=\sum _{j \in N_{i,sep}} \mathbf {F_{s,ij}} = \left\{ \begin{array}{ll} -k_{n}\delta \mathbf {\hat{x}_{ij}}, & \quad \delta <0\\ \textbf{0}, & \quad \textrm{otherwise}, \end{array}\right. \end{aligned}$$

where, $$N_{i,sep}$$ is the set of agents in the separation zone of agent *i*, i.e., $$\Vert x_{ij}\Vert \le r_{sep}$$, $$k_n$$ is the stiffness constant of the Hertzian contact force, $$\mathbf {\delta }=\left\| \mathbf {x_i}-\mathbf {x_j}\right\| -r_{sep}$$ is the separation between the two agents, $$\mathbf {\hat{x}_{ij}}=\frac{\mathbf {x_i}-\mathbf {x_j}}{\left\| \mathbf {x_i}-\mathbf {x_j}\right\| }$$ is a unit vector pointing away from agent *j* relative to agent *i.*$$d_{i}$$$$d_{j}$$ $$r_{sep}=((d_{i}+d_{j})/2)$$ is the radius of the separation zone (where, *d*_i_ and *d*_*j*_ are the diameters of the respective agents *i* and *j*), which, because of monodisperse size distribution among the prey, reduces to $$r_{sep}=d_{pr}$$.

The self-propulsion force begets the term “active matter” and is the force responsible for the movement of the agents in a desired direction. It could, otherwise, be defined as the will of the agent and is accountable for the non-equilibrium nature of active systems. In the present work, the prey agent has two states of activity: a cruising state and an escape state (see Eq. 4). The cruising state occurs in the absence of any danger (predator) in the vicinity, while the escape state requires the presence of a predator within the prey’s zone of detection $r_{d}$. The line of action of the force is in the direction of the current velocity when the prey is in cruising state, while that for a prey in escape state is in the direction away from the predator’s position^50^. Following literature that reports the escape behaviour of prey in the form of a sudden burst of motion^62,63^, the self-propulsion drive is implemented in terms of accelerations $\beta _{pr,e}$ and $\beta _{pr,c}$.

4 $$\begin{aligned} \mathbf {F_{p,pr}}=\left\{ \begin{array}{ll} m_{pr}\left( \beta _{pr,c} - \gamma \left\| \mathbf {v_{pr}}\right\| ^2 \right) \mathbf{\hat{v}_{pr}}, & \quad \textrm{cruising}\\ m_{pr}\left( \beta _{pr,e} - \gamma \left\| \mathbf {v_{pr}}\right\| ^2 \right) \mathbf{\hat{x}_{pr,e}}, & \quad \textrm{escape}, \end{array}\right. \end{aligned}$$

where, $\mathbf {v_{pr}}$ is the velocity of the prey agent *pr*, $\mathbf{\hat{x}_{pr,e}} = \frac{\mathbf {x_{pr}}-\mathbf {x_{pd}}}{\left\| \mathbf {x_{pr}}-\mathbf {x_{pd}}\right\| }$ is a unit vector in the direction of the shortest approach from the predator *pd* towards the prey agent *pr*, $\beta _{pr,c}$ and $\beta _{pr,e}$ are the cruising and escape accelerations, respectively, and $\gamma$ is the Rayleigh friction factor to prevent unbounded accelerations.

The alignment force is the cause of the flocking phenomena and is a force-based modification of the alignment rule proposed by Vicsek et al.^20^. Every agent attempts to align its velocity to that of its neighbours (see Eq. 5). The neighbourhood definition and associated interaction rules remain a highly debatable subject in collective dynamics and have been found to be highly species-dependent^24,63–65^. Therefore, in the absence of a proven generic neighbourhood definition, a metric distance-based model has been preferred in the current work over the kNN or the Voronoi neighbours approach, in the spirit of Vicsek alignment rules.

5 $$\begin{aligned} \mathbf {F_{c,pr}}=C_{v}d_{pr}\left( \phi _{pr}\mathbf {v_{surr,pr}}-\mathbf {v_{pr}}\right) , \end{aligned}$$

where, $\phi _{pr} = \frac{\sum _{j\in N_{pr,alg}} m_j}{\sum _{j\in N_{pr,alg}} m_j + m_{fluid}}$ is the mass fraction of live prey agents surrounding the agent *pr*, $\mathbf {v_{surr,pr}}=\frac{\sum _{j\in N_{pr, alg}}m_{j}W_{pr,j}\mathbf {v_j}}{\sum _{j\in N_{pr,alg}}m_{j}W_{pr,j}}$ is the weighted average velocity of the live prey neighbours in the alignment zone of agent *pr* ($N_{pr, alg}$ is the set of such neighbours), $d_{pr}$ is the characteristic dimension of the agent (here, the diameter), *m*_*fluid*_ is the mass of the fluid surrounding agent *pr*, and $C_{v}$ is the strength of alignment among the prey. A Gaussian weight function $W_{pr,j}$ centred at agent *pr* and depending on distance $\left\| \mathbf {x_{pr}}-\mathbf {x_{j}}\right\|$ is employed. The weight function has a narrow RMS width, such that the alignment interactions are localised, reducing the effective number of influential neighbours. An orientational white noise $\zeta$ with zero mean and standard deviation $\sigma _{\zeta }$ is superposed on the direction of the alignment force as shown in Eq. 6, similar to Agrawal & Mahapatra^66^.

6 $$\begin{aligned} \mathbf {F_{c,pr}} = \Vert \mathbf {F_{c,pr}}\Vert \left( \begin{array}{c} \cos (\theta _{c,pr}+\zeta ) \\ \sin (\theta _{c,pr}+\zeta )\end{array} \right) , \end{aligned}$$

where, $\theta _{c,pr}$ is the angle, the computed alignment force for agent *pr*, makes with the x-axis.

The attraction force is a long-range pairwise force that acts to drive any prey towards live conspecifics within its attraction zone. The functional form of the attraction force (defined by Warburton & Lazarus^67^) is such that the farther a prey is from the nearest conspecific, the more vigorous is the drive to close the distance (see Eq. 7). As the distance between the prey decreases, the attraction force gradually reduces until the conspecific is within the alignment zone of the agent.

7 $$\begin{aligned} \mathbf {F_{a, pr}} = \upsilon _{a} \sum _{j\in N_{pr,atr}}\left( 1-\left( 1-\frac{\left\| \mathbf {x_{pr}}-\mathbf {x_{j}}\right\| }{r_{atr}}\right) ^2\right) ^{1/2} \end{aligned}$$

where, $\upsilon _{a}$ is the strength of the attraction force, $r_{atr}$ is the radius of the attraction zone, and $N_{pr,atr}$ is the set of live prey neighbours in the attraction zone of agent *pr*. The value of $\upsilon _{a}$ is set such that in the absence of predators, the prey form a single cohesive group; however, high self-propulsion forces due to predatory threats can disrupt the cohesion and result in splitting of the group.

The frictional force $\mathbf {F_f}$, as the name suggests, acts opposite to the direction of motion of the agent. The frictional drag experienced by the organism due to the relative velocity with respect to the surrounding medium can be approximated, following Viscido et al.^68^ as Eq. 8. A stationary fluid medium has been assumed in this study.

8 $$\begin{aligned} \mathbf {F_{f,pr}} = -\frac{1}{2}\rho c_{f}A\left\| \mathbf {v_{pr}}\right\| \mathbf {v_{pr}}, \end{aligned}$$

where, $c_f$ is the drag coefficient, *A* is the surface area of the agent in contact with the fluid, and $\rho$ is the density of the surrounding medium.

### Predator agent

The governing equation for a predator agent *pd* consists of a self-propulsion term, a friction drag, and a short-range pairwise separation term (see Eq. 9).

9 $$\begin{aligned} m_{pd}\mathbf {\ddot{x}_{pd}} = \mathbf {F_{s,pd}} + \mathbf {F_{f,pd}} + \mathbf {F_{p,pd}}, \end{aligned}$$

where, the right-hand side represents the sum of the pairwise separation force $\mathbf {F_{s,pd}}$, the frictional force $\mathbf {F_{f,pd}}$, and the self-propulsion force $\mathbf {F_{p,pd}}$. $m_{pd}$ and $\mathbf {x_{pd}}$ represent the mass and the position of the predator agent, respectively. The current work assumes the predator agent to be alive throughout the simulation. The zone-based classification for the predator constitutes a capture zone $R_c$ and a detection zone $R_d$ (see Fig. 7(b)). The predator can scan the live prey in the detection zone before selecting a target. Any target prey which enters the capture zone is captured, changing the $\mu$ from 1 to 0 for that prey agent (more details in subsequent paragraphs).

The pairwise separation and the frictional forces for the predator agent have the same functional form as that of the prey agents. The self-propulsion force, on the other hand, is distinctly different and closely connected to the states of the predator. The predator agent can have three states: satisfied state, refocus state and pursuit state. The satisfied state occurs when the predator successfully captures its target prey, while the refocus state occurs in case of failure to capture the target. The propulsion force, in both the aforementioned states, acts in the direction of the current velocity $\left( \mathbf{\hat{v}_{pd}}\right)$, similar to that of the prey, while in the pursuit state, the predator propels itself in the direction of the shortest distance towards the target prey (see Eq. 10). Similar to that of the prey, the self-propulsion drive of the predator has been modelled using accelerations, citing reports in extant literature on a short-lived but high acceleration during hunting^16^.

10 $$\begin{aligned} \mathbf {F_{p,pd}}=\left\{ \begin{array}{ll} m_{pd}\left( \beta _{pd,sr} - \gamma \left\| \mathbf {v_{pd}}\right\| ^2 \right) \mathbf{\hat{v}_{pd}}, & \quad \mathrm{satisfied/refocus}\\ m_{pd}\left( \beta _{pd,h} - \gamma \left\| \mathbf {v_{pd}}\right\| ^2 \right) \mathbf{\hat{x}_{pd,h}}, & \quad \textrm{pursuit}, \end{array}\right. \end{aligned}$$

where $\beta _{pd,sr}$ and $\beta _{pd,h}$ are the satisfaction/refocus and the pursuit accelerations of the predator, respectively, $\gamma$ is the Rayleigh’s friction factor to prevent unbounded acceleration, $\mathbf {v_{pd}}$ is the velocity of the predator and $\mathbf{\hat{x}_{pd,h}}$ is the direction from the predator towards the target prey.

**Fig. 8:**
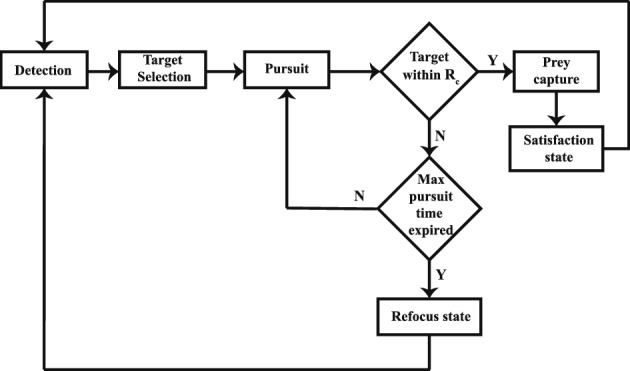
A flowchart depicting the process of predation used in the model.

The predation process, as defined in one of our earlier works^50^, consists of multiple stages: a) detection stage, b) target selection stage, c) pursuit stage, and d) satisfaction/refocus stage. The process has been illustrated in the form of a flowchart in Fig. 8. In the detection stage, the predator detects all live prey in its vicinity (i.e., its detection zone $r \le R_{d}$ sans the blind spot). In the next stage, the predator selects a target prey based on its attack preference (either the nearest prey or the most central prey). Once a target prey is selected, the predator starts pursuing the target until a maximum allowed pursuit time $\tau _{P}$ expires (pursuit stage). If the predator is able to capture the target prey (i.e., if the target prey moves into the capture zone of the predator), it enters a satisfaction stage for some time $\tau _S$, where it cruises around without hunting. On the contrary, if the predator is unsuccessful in capturing the target prey within the stipulated time period $\tau _P$, it enters a refocus stage (i.e., it takes time $\tau _R$ to collect its bearings). After the satisfaction time $\tau _S$ or refocus time $\tau _R$ has passed, the predator actively starts detecting potential target prey again. The above predation process has been reported by Romenskyy et al.^16^ and used a priori by Demšar et al.^45^.

In nature, the predator selects its target prey based on a myriad of environmental factors, such as the presence of prey feeding spots, potential obstacles, ambush-friendly foliage, and competing species, to name a few, as well as internal factors, such as the ability to camouflage, the proclivity for group hunting, and accrued experiential learning, apart from the sensory cues^43^. In the current work, visual cues are assumed to be the only source of information, and there are two possible choices for a target: the nearest prey (hereby called NP) or the most central prey (hereby called MCP)^44^. In an NP attack (see Fig. 7(c)), the predator attacks the live prey nearest to its position (within its field of view), while in an MCP attack (see Fig. 7(d)), the predator attacks the live prey closest to the algebraic centre of the visible group. As the present work focuses on two independently attacking predators, three cases have been considered through combinatorics of the two target selection methods. The first case consists of all the predators pursuing their respective nearest live prey (hereby called the purely NP strategy). In the second case, every predator has an equal probability of choosing the nearest prey (NP) or the most central prey (MCP) in the target selection stage of each hunting cycle (NP-MCP combination strategy). A deterministic version of the NP-MCP combination strategy has also been looked into, where one of the predators attacks the nearest prey, and the other predator attacks the most central prey throughout the simulation time. The results from the stochastic and the deterministic variants of the NP-MCP strategy are compared in Fig. S6 of Supplementary Information. The third and final case considers the predators choosing their respective most central prey (MCP) as the target (purely MCP strategy).

### Simulation details

The system under observation constitutes a group of $N=506$ prey agents and two predators in a square periodic 2D domain of side *L*. Both the prey and the predator agents are assumed to be soft disks and can occupy off-lattice positions. The physical parameters, such as size and mass of the prey agent, are taken from the works of Viscido et al.^26,68^ on giant danio (*Devario aequipinnatus*). The predators’ mass is much higher (around $16\times$) than the prey, so that they have lower manoeuvrability, as has been reported in literature^11^. The predators capture the prey; however, they consume a negligible portion of it such that the predator mass remains unchanged. The forces acting on each of the agents are calculated at every time step, and the velocities and displacements are updated synchronously using the Velocity-Verlet algorithm. The simulations explore the parameter space for the non-dimensional prey coordination strength $\chi =\frac{C_v d_{pr}v_{pr}}{m_{pr}\beta _{pr,e}} \sim \frac{C_v d_{pr}}{m_{pr}}\sqrt{\frac{d_{pr}}{\beta _{pr,e}}}$ (the relative strength of the alignment force compared to the self-propulsion force in the escape state), the relative angle of the predators’ attack $\Omega _P$, and the different attack strategies (see the previous subsection for more details). The self-propelling accelerations of the agent are chosen such that the velocities of the agents are close to realistic values reported in literature^26^. The vision cone of the agents is chosen from extant literature as well^45,69^. The parameters related to length have been non-dimensionlised against the characteristic length of the prey (diameter $d_{pr}$). Time is non-dimensionalised as $\tau =\frac{t}{\sqrt{d_{pr}/\beta _{pr,e}}}$ against the time taken by a single evading prey agent to traverse its own length in a ballistic fashion (only the self-propulsion force acts on it), while the speed is non-dimensionalised against the corresponding speed of the ballistic prey agent $v_0 = \sqrt{d_{pr}\beta _{pr,e}}$. The parameters affecting the speed of the prey are carefully set such that the predators’ speed is always greater than that of the prey (as evidenced in literature^11^). The detailed list of parameters and their chosen values are tabulated in Table S3 of Supplementary Information. Simulations have also been carried out to understand the effect of domain size on prey behavioural response, and the results are reported in Figs. S7a and S7b of Supplementary Information. At least fifty realisations have been run with different starting positions of the predators with respect to the prey group, and the reported results are an average over all the realisations unless specified otherwise. The simulations have been carried out using an in-house C++ code, and the data has been processed and plots generated using MATLAB and *seaborn* package in Python 3.8.

## Supplementary Information

Supplementary Information 1.

Supplementary Information 2.

## Data Availability

The datasets for the figures along with the codes used to generate the data and the figures are available at https://github.com/s-m-sys/async_multi_predator.
