# Supplementary material for: Behavioural response of prey to repeated attacks by non-coordinating predators
Source: Sci Rep. 2025 Jul 2;15:22977. doi: 10.1038/s41598-025-05946-6 (PMC12214627; doi:10.1038/s41598-025-05946-6)
Supplement: Supplementary file 1 — Supplementary Information 1. [file 41598_2025_5946_MOESM1_ESM.pdf]

Supplementary Information

Behavioural response of prey to repeated attacks by non-coordinating predators

Siddhant Mohapatra[1] **and Pallab Sinha Mahapatra**[1,*]

[1]Department of Mechanical Engineering, Indian Institute of Technology Madras, Chennai, Tamil Nadu, India 600036.
[*]pallab@iitm.ac.in

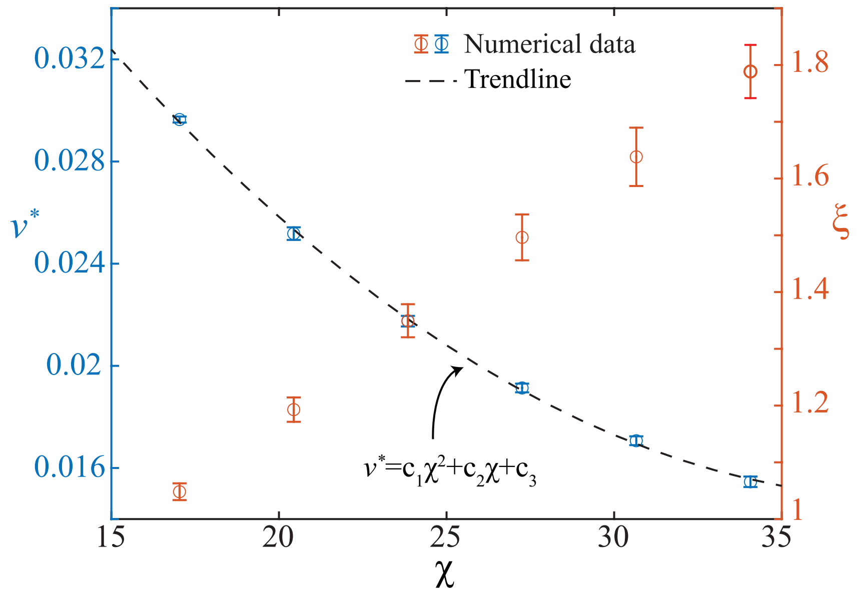

Figure S1. ***Decay of prey speed with prey coordination***: The average non-dimensional speed of the prey v^* (left axis; blue) and the ratio ξ of average predator speed to average prey speed (right axis; red) are plotted against the prey coordination strength χ. v^* follows a quadratic monotonically decreasing relation with χ as evidenced by the trend line (dashed black line); the coefficients of the fit with 95% CI are as follows: $c_1 = 3.038 \times 10^{-5}\left(2.322 \times 10^{-5}, 3.754 \times 10^{-5}\right)$, $c_2 = -2.373 \times 10^{-3}\left(-2.741 \times 10^{-3}, -2.006 \times 10^{-3}\right)$, $c_3 = 0.06115\,(0.05662, 0.06567)$.

SI-1: Determining the nature of the decay in live prey with time

One approach to characterising the long-term dynamics of a predator-prey system is by tracking the number of live prey with time. Here, the temporal decay in the fraction of live prey in the system N_l (normalised against the total number of prey agents in the system, i.e., $N = 506$) is portrayed under differing cases of prey coordination strength $\chi = \frac{C_v d_{pr}^{3/2}}{m_{pr}\sqrt{\beta_{pr,e}}}$ and predators' attack strategy. Figure S2(a) illustrates the possible fitting relations for N_l with non-dimensional time $\tau = t/\sqrt{d_{pr}/\beta_{pr,e}}$ at $\chi = 17$, $\chi = 24$, and $\chi = 31$, when the predators pursue the nearest live prey in their neighbourhood (also known as the purely NP strategy). The details of the fitting parameters and the correlation coefficients are tabulated in Table S1. Figure S2(b), on the other hand, delineates the possible fitting relations for N_l with time τ at the same χ values when the predators have an equal probability of choosing between the nearest prey and the most central prey in their vicinity at the beginning of each predation cycle (also known as the stochastic NP-MCP combination strategy). The details of these fitting parameters and correlation coefficients are tabulated in Table S2.

Irrespective of the prey coordination strength χ and the type of attack strategy followed by the predators, the exponential fit gives poor goodness-of-fit for N_l (see Figs. S2(a) and S2(b)), between $\tau = 5 \times 10^3$ and $\tau = 2 \times 10^5$. On the contrary, the linear fit provides comparatively promising prospects in that regard. Generally speaking, the quadratic fit gives the best fit for all the cases mentioned above. However, in cases such as predators following purely NP strategy at $\chi = 31$ (see Fig. S2(a)) and NP-MCP combination strategy at $\chi = 24$ and $\chi = 31$ (see Fig. S2(b)), the linear fit for N_l suffices as a good enough fit. Therefore, it can be concluded that the underlying process for N_l for the above cases is linear in time, while for the remaining cases, the underlying process for N_l can be thought of as quadratic in time (refer to Tables S1 and S2).

Table S1. Fitting parameters for the fraction of live prey in the system N_l against non-dimensional time τ; both predators follow the nearest prey attack strategy (purely NP strategy).

χ	Nature of fit	Regression relation	Correlation (r)
17	Exponential	$N_l = 1.0551e^{-2.7248\times10^{-6}\tau}$	−0.9982727
	Linear	$N_l = 0.93967 - 1.4621 \times 10^{-6}\tau$	−0.992931
	Quadratic	$N_l = 1.0018 - 2.16 \times 10^{-6}\tau + 1.3543 \times 10^{-12}\tau^2$	
24	Exponential	$N_l = 1.1653e^{-8.135\times10^{-6}\tau}$	−0.9892865
	Linear	$N_l = 0.9693 - 4.039 \times 10^{-6}\tau$	−0.9984386
	Quadratic	$N_l = 1.0061 - 5.0012 \times 10^{-6}\tau + 4.5738 \times 10^{-12}\tau^2$	
31	Exponential	$N_l = 1.1067e^{-1.0625\times10^{-5}\tau}$	−0.9929321
	Linear	$N_l = 0.99859 - 6.4235 \times 10^{-6}\tau$	−0.9998129
	Quadratic	$N_l = 1.0087 - 6.9065 \times 10^{-6}\tau + 4.2734 \times 10^{-12}\tau^2$	

Table S2. Fitting parameters for the fraction of live prey in the system N_l against non-dimensional time τ; the predators follow the NP-MCP combination strategy.

χ	Nature of fit	Regression relation	Correlation (r)
17	Exponential	$N_l = 0.97813e^{-1.2795\times10^{-6}\tau}$	−0.9997189
	Linear	$N_l = 0.89101 - 6.7588 \times 10^{-7}\tau$	−0.9820754
	Quadratic	$N_l = 0.97967 - 1.178 \times 10^{-6}\tau + 4.8292 \times 10^{-13}\tau^2$	
24	Exponential	$N_l = 1.1228e^{-6.0379\times10^{-6}\tau}$	−0.9908953
	Linear	$N_l = 0.98418 - 3.3229 \times 10^{-6}\tau$	−0.9992048
	Quadratic	$N_l = 1.0065 - 3.863 \times 10^{-6}\tau + 2.2902 \times 10^{-12}\tau^2$	
31	Exponential	$N_l = 1.1658e^{-1.0354\times10^{-5}\tau}$	−0.9861219
	Linear	$N_l = 0.99239 - 5.4228 \times 10^{-6}\tau$	−0.9995274
	Quadratic	$N_l = 1.0104 - 6.0947 \times 10^{-6}\tau + 4.3854 \times 10^{-12}\tau^2$	

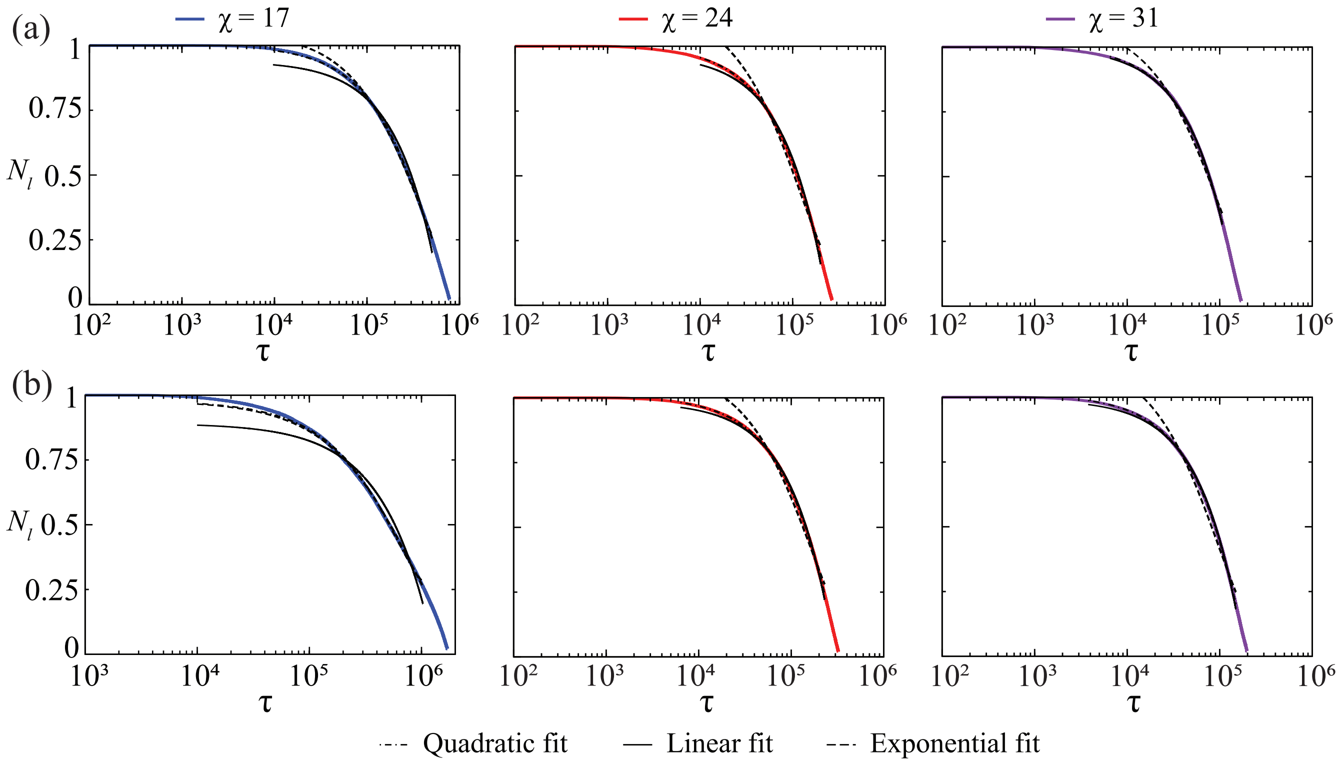

Figure S2. ***Comparison of different fitting functions for live prey data***: The figure illustrates the variation of live prey fraction in the system N_l with non-dimensional time τ and the linear, exponential and quadratic fits of the data (solid, dashed, dash-dot black lines, respectively). The blue, red, and violet curves represent prey coordination strengths of $\chi = 17$, $\chi = 24$ and $\chi = 31$, respectively. The predators can pursue either the respective nearest live prey in their neighbourhood (purely NP strategy; shown in panel (a)), or choose with equal probability between the nearest live prey and the most central live prey in their neighbourhood at the beginning of the predation cycle (NP-MCP combination strategy; shown in panel (b)).

Table S3. ***Simulation parameters***: The parameters used in the simulations (including supplementary information) are listed below. The first section lists the parameters that have been used to define the numerical model and are kept unchanged across all simulations, while the second section lists the parameters that have been varied in the study.

Parameter	Definition	Values
**Sec. I**	**Constant parameters**	
$\beta_{pd,h}$	Pursuit acceleration of predator	$0.08[ms^{-2}]$
$\beta_{pd,sr}$	Satisfaction/refocus acceleration of predator	$0.5\beta_{pd,h}$
$\beta_{pr,e}$	Escape acceleration of prey	$15[ms^{-2}]$
$\beta_{pr,c}$	Cruising acceleration of prey	$0.5\beta_{pr,e}$
d_{pd}	Characteristic dimension of predator	$4d_{pr}$
d_{pr}	Characteristic dimension of prey	$0.0272[m]$
γ	Rayleigh friction factors for prey and predator	$0.1[m^{-1}]$
k_n	Stiffness constant of Hertzian contact force for separation	$2\times 10^4[Ns^{-1}]$
m_{pd}	Mass of the predator agent	$16m_{pr}$
m_{pr}	Mass of the prey agent	$1.7\times 10^{-3}[kg]$
R_d	Radius of detection zone of predator	$5d_{pd}$
R_c	Radius of capture zone of predator	$0.5(d_{pd}+d_{pr})$
r_{alg}	Radius of alignment zone of prey	$5d_{pr}$
r_{atr}	Radius of attraction zone of prey	$20d_{pr}$
r_d	Radius of detection zone of prey	$20d_{pr}$
r_{sep}	Radius of separation zone of prey	d_{pr}
ρ	Density of the surrounding medium	$1000[kgm^{-3}]$
σ_ζ	Standard deviation of white orientational noise	0.3
τ_P	Maximum pursuit time for predator	470
τ_R	Refocus time for predator	$0.25\tau_P$
τ_S	Satisfaction time for predator	$0.5\tau_P$
θ_b	Blind angle for prey and predator	$\pi/3$
υ_a	Strength of the attraction force	$10^{-6}[N]$
**Sec.II**	**Variable parameters**	
χ	Non-dimensional coordination coefficient	$17-31$
L	Length of the domain	$\{92d_{pr}, 276d_{pr}, 368d_{pr}, 552d_{pr}, 920d_{pr}\}$
N	Number of prey agents	$\{100, 256, 361, 506, 625, 756\}$
N_p	Number of predator agents	$\{1, 2, 5\}$

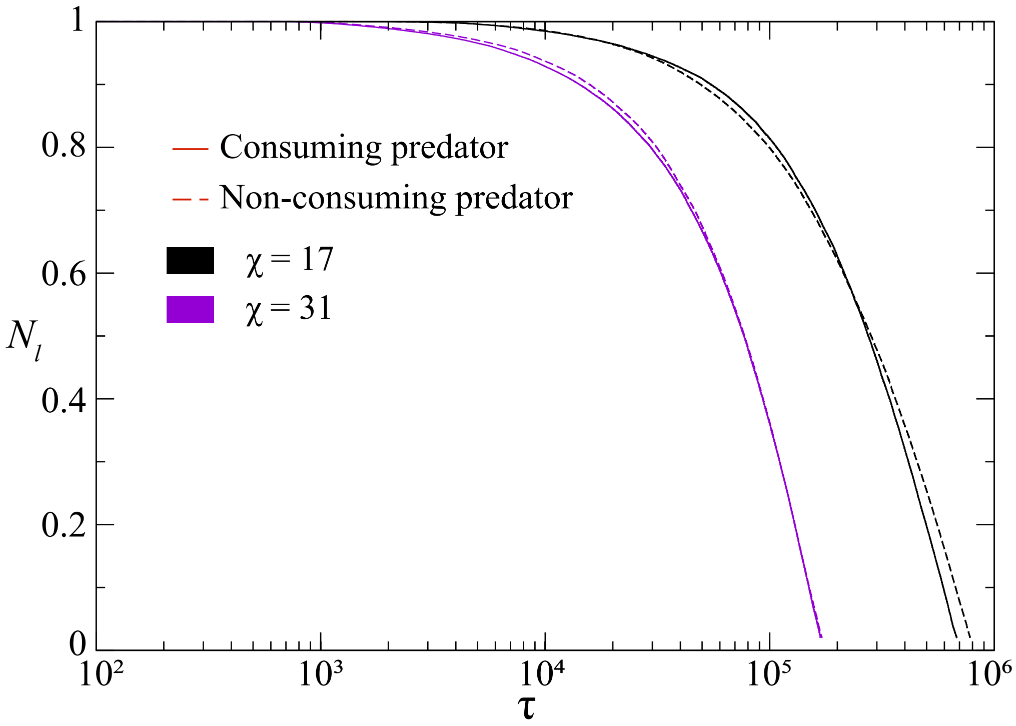

Figure S3. ***Comparison of a consuming and a non-consuming predator***: The temporal variation of the fraction of live prey N_l (normalised against the initial number of prey in the system, i.e., $N = 506$) has been compared for a consuming predator (the predator consumes the captured prey; therefore, the captured prey are removed from the system; represented by solid black line for $\chi = 17$ and solid violet line for $\chi = 31$) and a non-consuming predator (the predator consumes a minuscule part of the captured prey; therefore, captured prey act as obstacles/passive agents and are acted upon only by pairwise separation force and the fluid friction force; represented by dashed black line for $\chi = 17$ and dashed violet line for $\chi = 31$). A long-term temporal study of the two systems displays close to identical behaviour for different prey coordination strength χ. (Note: Both the predators prefer attacking their respective nearest live prey (purely NP strategy).)

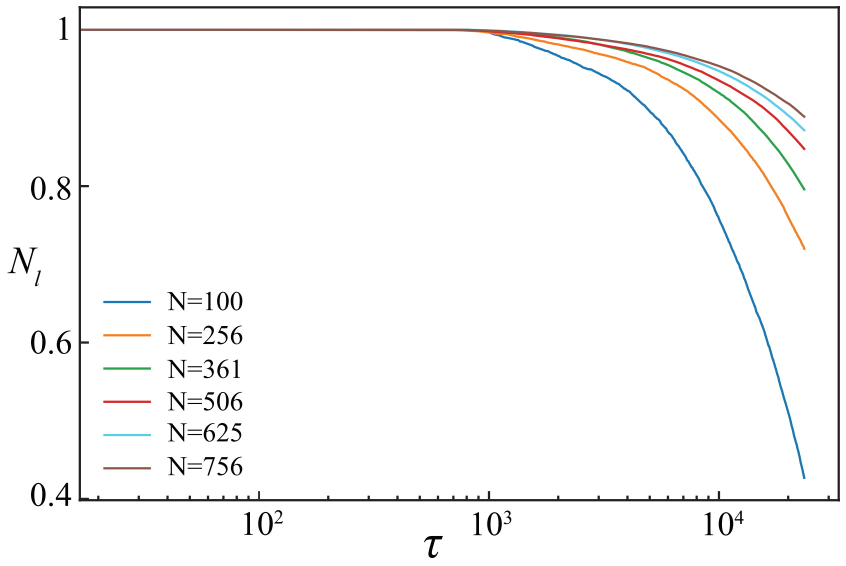

Figure S4. ***Effect of initial prey group size on survival probability***: The fraction of live prey agents N_l is plotted against simulation time $\tau = t/\sqrt{d_{pr}/\beta_{pr,e}}$. The difference in the slope (representing the prey capture rate) is visually apparent, with higher initial prey numbers (higher N) leading to lower capture rates. This observation holds well with the many-eyes hypothesis, which accentuates the relative safety of an organism in larger groups of conspecifics due to increased vigilance against predation. (Note: The data represented are averaged over fifty realisations. Prey coordination strength χ is set to 31, and both the predators follow the nearest prey hunting strategy. N_l at any time τ is the ratio of live agents at time τ to that at $\tau = 0$. τ is the non-dimensional time, calculated by dividing a normalisation factor equivalent to the time taken by a prey agent to accelerate its own length d_{pr} ballistically. Domain length $L = 92d_{pr}$.)

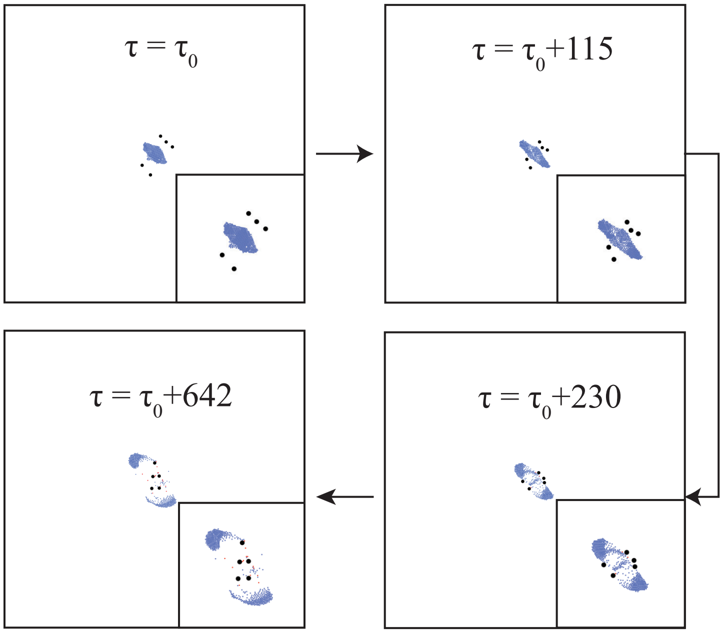

Figure S5. ***Five independent predators attacking a prey group***: The prey response in the case of five independently hunting predators is illustrated in the form of snapshots of the time progression of the system. A split-and-escape prey response is observed for this initial system state; however, before the constriction in the middle can completely separate, the predators are able to pursue and capture a significant number of prey. (Note: Live and captured prey agents are coloured blue and red, respectively, while the predators are coloured black. The domain size is $L = 368d_{pr}$. The zoomed-in view is shown in the bottom right corner of each snapshot.)

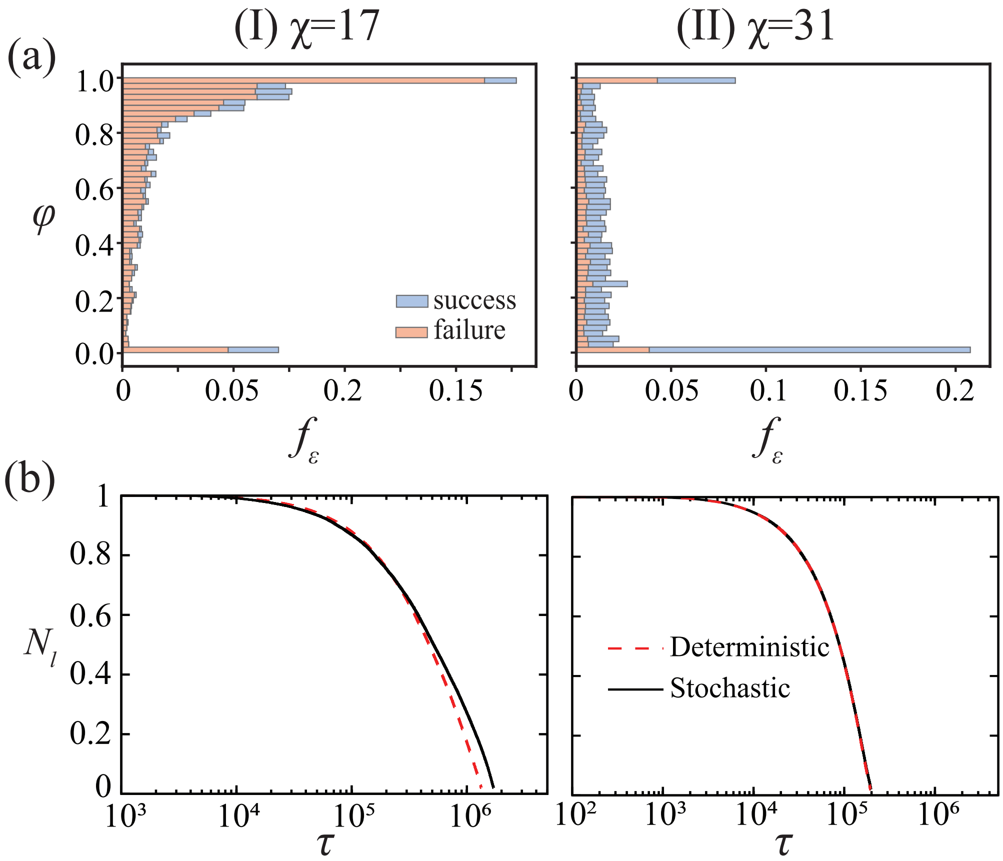

Figure S6. ***Comparison of predators' deterministic NP-MCP and stochastic NP-MCP combination attack strategies***: In the case of the deterministic NP-MCP combination attack strategy, one predator is set to attack the nearest prey (NP), while the other is set to attack the most central prey (MCP) throughout the simulation time. On the other hand, in the case of the stochastic NP-MCP attack strategy, it is equally probable for each predator to select between the NP and the MCP options for each predation cycle. Row (a) portrays the fraction f_ε of pursuits on prey group sizes φ that end up in success (blue) or failure (orange) for the deterministic case. The overall probability of successful prey capture for the deterministic case is $\approx 16\%$ and $\approx 69\%$ against $\approx 17\%$ and $\approx 68\%$ for the stochastic variant at prey coordination strengths $\chi = 17$ (column I) and $\chi = 31$ (column II), respectively. Row (b) compares the long-term behaviour of the system by presenting the decay in live prey fraction N_l with non-dimensional time τ. The curves are similar for both the stochastic (solid black line) and the deterministic (dashed red line) cases, indicating almost identical dynamics. (Note: φ and N_l are normalised against the total number of prey agents in the system initially N; here, $N = 506$. The domain size $L = 92d_{pr}$.)

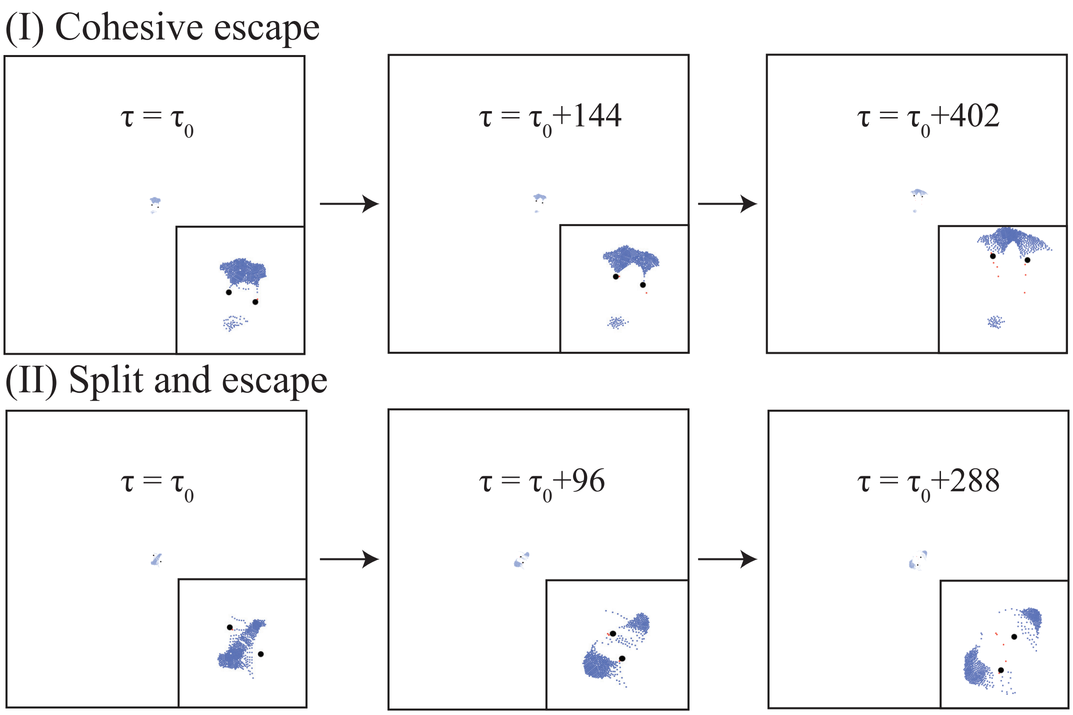

Figure S7a. ***Effect of domain size on predators' performance - qualitative comparison***: The effect of domain size on the prey response to predator attacks has been demonstrated in this figure, with the top panel showcasing the cohesive escape response and the bottom panel illustrating the split and escape response. The prey response is compared qualitatively for domain sizes $L = 92d_{pr}$ (used in the manuscript) and $L = 920d_{pr}$, keeping all other parameters unchanged. Similar prey behaviour is observed, irrespective of domain size. The dimensions shown in the figure are to scale, and the live and captured prey are coloured blue and red, respectively, while the predators are coloured black. (Note: $\tau = t\sqrt{\frac{\beta_{pr,e}}{d_{pr}}}$ is the normalised time. The zoomed-in view is shown in the bottom right corner of each snapshot.)

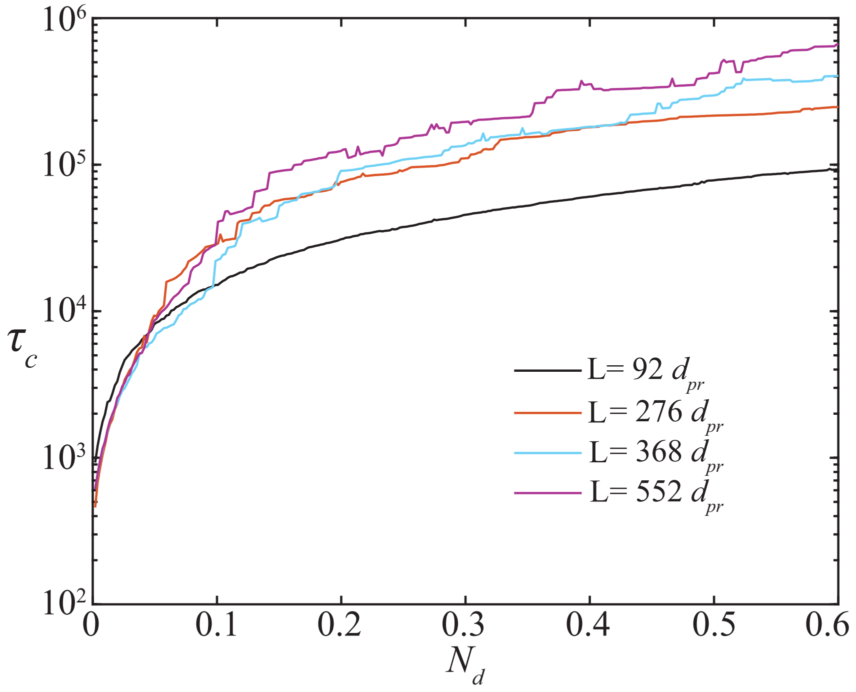

Figure S7b. ***Effect of domain size on predators' performance - long-term behaviour***: The average time taken $\tau_c = t_c/\sqrt{d_{pr}/\beta_{pr,e}}$ to capture a certain fraction of prey N_d (normalised against the total number of prey agents in the system initially, here, $N = 506$) is plotted for different domain sizes L. As the domain size increases, it takes slightly longer for the predators to capture the same fraction of prey due to increased prey fragmentation and, thereby, increased search time before encountering live prey. (Note: All the curves represent an average over at least fifteen realisations; coordination strength χ of the prey is set at 31; both predators follow the nearest prey (NP) hunting strategy.)
